# Maternity health professionals’ perspectives of cord clamp timing, cord blood banking and cord blood donation: a qualitative study

**DOI:** 10.1186/s12884-020-03102-8

**Published:** 2020-07-16

**Authors:** Lisa Peberdy, Jeanine Young, Debbie Massey, Lauren Kearney

**Affiliations:** 1 Clinical Nursing – Maternal, Child and Family Health, The University of the Sunshine Coast, 90 Sippy Downs Drive, Sippy Downs, Queensland 4556 Australia; 2 The University of the Sunshine Coast, 90 Sippy Downs Drive, Sippy Downs, Queensland 4556 Australia; 3 Southern Cross University, Gold Coast Airport, Terminal Dr, Bilinga, Queensland 4225 Australia

**Keywords:** Cord clamping, Cord blood banking, Cord blood donation, Knowledge, Perspectives, Practice, Midwives, Obstetricians

## Abstract

**Background:**

Parents today have several options for the management of their infant’s cord blood during the third stage of labour. Parents can choose to have their infant’s cord clamped early or to have deferred cord clamping. If the cord is clamped early, cord blood can be collected for private cord blood banking or public cord blood donation for use later if needed. If cord clamping is deferred, the placental blood physiologically transfuses to the neonate and there are physiological advantages to this. These benefits include a smoother cardiovascular transition and increased haemoglobin levels while not interfering with the practice of collecting cord blood for gases if needed. The aim of this study is to explore Australian maternity health professionals’ perspectives towards cord clamp timing, cord blood banking and cord blood donation.

**Methods:**

Fourteen maternity health professionals (midwives and obstetricians) from both private and public practice settings in Australia participated in semi-structured interviews either in person or by telephone. Interviews were transcribed and data analysed using thematic analysis.

**Results:**

Overall there was strong support for deferred cord clamping, and this was seen as important and routinely discussed with parents as part of antenatal care. However, support did not extend to the options of cord blood banking and donation and to routinely informing parents of these options even when these were available at their birthing location.

**Conclusion:**

Formalised education for maternity health professionals is needed about the benefits and implications of cord blood banking and cord blood donation so that they have the confidence to openly discuss all options of cord clamp timing, cord blood banking and cord blood donation to facilitate informed decision-making by parents.

## Background

Parents today have several available options for the management of their infant’s umbilical cord blood during the third stage of labour: physiological transfusion of blood from placenta to newborn; banking of the cord blood for private use; or donation of cord blood for public banks to be used for therapeutic use in conditions of the blood and the immune system [1].

The timing of cord clamping after birth has been controversial for decades, although in recent years there has been a growing interest from health professionals and professional bodies in the physiology of placental transfusion and the optimal time to clamp the umbilical cord. Interdisciplinary international guidelines now recommend deferred cord clamping for a minimum of 1 min after the birth of the infant [2, 3]. This renewed interest and focus on cord clamping is the result of research surrounding the benefits to the infant associated with the timing of cord clamping at birth [4, 5]. These include a more stable cardiovascular transition [6], increased haemoglobin level at birth, increased iron stores for up to 6 months of age [4], improved fine motor and social domain scores at age 4 years [7], and no interference with the collection of valid cord blood gas samples [8].

Advances in scientific knowledge and the introduction of new technologies into the clinical arena increasingly challenge health professionals’ knowledge and traditional practices. Knowledge of the value of full placental transfusion and the unique properties of cord blood stem cells could be argued to be the foundations upon which health professionals should develop their practices relating to third stage labour management, and the options that are subsequently provided to parents. In addition, the timing of cord clamping impacts the volume of blood for collection and banking purposes [9]. Several studies have investigated maternity health professionals’ knowledge, attitudes and practices pertaining to third stage labour options for parents internationally [10–13]. However, there is a paucity of knowledge regarding health professionals’ knowledge, perspectives and practices towards third stage labour options in the Australian context. Therefore, a gap exists in understanding health professionals’ knowledge of, and perspectives towards, current third stage labour options for parents and their practice of informing parents of these options. An explanatory mixed methods study involving sequential data collection in two phases (a cross-sectional survey and interviews) was recently conducted in Australia to explore maternity health professionals’ knowledge, perspectives, self-reported practices and their perspectives, of cord clamp timing, cord blood banking and cord blood donation for term infants. The survey study will be reported in detail, elsewhere.

This study described herein aimed to explore Australian health professionals’ perspectives of cord clamp timing, cord blood banking and cord blood donation for term infants.

## Methods

### Design

A qualitative, descriptive study design using semi-structured interviews conducted face-to-face or by telephone was used to gather in-depth views and perspectives [14] of maternity health professionals pertaining to parental third stage of labour options. The study is reported according to the Consolidated Criteria for Reporting Qualitative Health Research (COREQ) guidelines [15].

### Recruitment

The primary recruitment method was through the Australian College of Midwives and the Queensland Maternal and Neonatal Network e-bulletin announcements. The secondary recruitment method was through the placement of information regarding the study and survey online-link on two University social media sites, and through email and postal invitations to private practice obstetricians throughout Queensland, Australia.

Selection criteria for study participants included maternity health professionals who had provided antenatal and intrapartum care to pregnant women in Australia within the last 5 years and were registered with Australian Health Practitioner Regulation Agency (AHPRA). This allowed for a wide variety of views and experiences in different health care settings across Australia.

### Participants

Potential participants who nominated their interest in interview participation following the survey study (*n* = 14/129; 11%) were contacted by the researcher and given a Participant Information Sheet and written consent form. Fourteen participants (11 midwives, 3 obstetricians) completed the consent form and agreed to be interviewed. Participants were unknown to the researcher prior to commencement of the study. Participant mean range of length of clinical practice was 17.2 years. Participant characteristics (occupation, mode of interview, practice setting, cord blood banking option available in practice setting) are displayed in Table 1.

**Table 1:** Participant characteristics

Occupation	State	Method	Facility	Banking option
Midwife	QLD	Phone	Public Hospital; Regional	Private
Midwife	QLD	Phone	Public Hospital: Regional	Private
Midwife	QLD	Phone	Public Hospital; Regional	Private
Midwife	QLD	Phone	Public Hospital; Regional	Private
Midwife	QLD	Phone	Public Hospital; Metro	Private and Public Cord Blood Bank
Midwife	QLD	Face to Face	Private Hospital/Clinic; Metro	Private
Midwife	QLD	Phone	Public Hospital; Regional	Private
Midwife	NSW	Phone	Public Hospital/Community Setting; Regional	Private
Midwife	NSW	Phone	Public Hospital; Regional	Private
Midwife	VIC	Phone	Private Hospital; Regional	Private
Midwife	WA	Phone	Public Community Setting; Metro	Private
Obstetrician	QLD	Face to Face	Private Hospital; Metro	Private
Obstetrician	QLD	Phone	Private Hospital; Regional	Private
GP Obstetrician	QLD	Phone	Public Hospital; Regional	Private

*Legend*: *GP* General Practitioner /obstetrician, *NSW* New South Wales, *QLD* Queensland, *VIC* Victoria

### Data collection

Interviews were conducted by the primary author, a female midwife and doctoral candidate. Interviews were conducted between April and June 2018 and ranged from 15 to 59 min in length. Most interviews (*n* = 12/14) were conducted via telephone due to participant locations around Australia. Two interviews were conducted in person. Interviews were recorded and transcribed. The interview guide contained 23 semi-structured questions. The semi-structured format guided the interview process and ensured the required information was covered by the participants [16]. The interview questions are available as a Supplementary File. Participant confidentiality was maintained using pseudonyms in interview transcripts. Interviews continued until data saturation was achieved [17]. Participants were offered the opportunity to review the transcript of their interview however none requested this.

### Data analysis

Thematic analysis was used by the primary author for the qualitative data analysis, following the six steps described by Braun and Clarke (2006). This included becoming familiar with the data collected, systematically generating codes of the transcripts, searching for and summarising codes into meaningful themes pertinent to the research question, reviewing the themes through the development of a thematic map, and defining and naming the themes [18]. To enhance rigour, a range of strategies was implemented. These included a robust review of the research design and data analysis by all authors. Robust discussions ensured that the findings were presented from a conscious, transparent perspective of the phenomenon that emerged from the research. Frequent sessions were conducted to discuss theme development to test interpretation of the data and identify individual bias and interpretation with all authors. An important strategy to enhance credibility is to incorporate the appropriate operational measures [19]. The use of a range of participants helped to promote credibility. This study included midwives and obstetricians from community, public and private hospital settings which facilitated the verification of experiences and perceptions to construct a rich picture on the phenomena of interest being investigated.

## Results

**Fig. 1:**
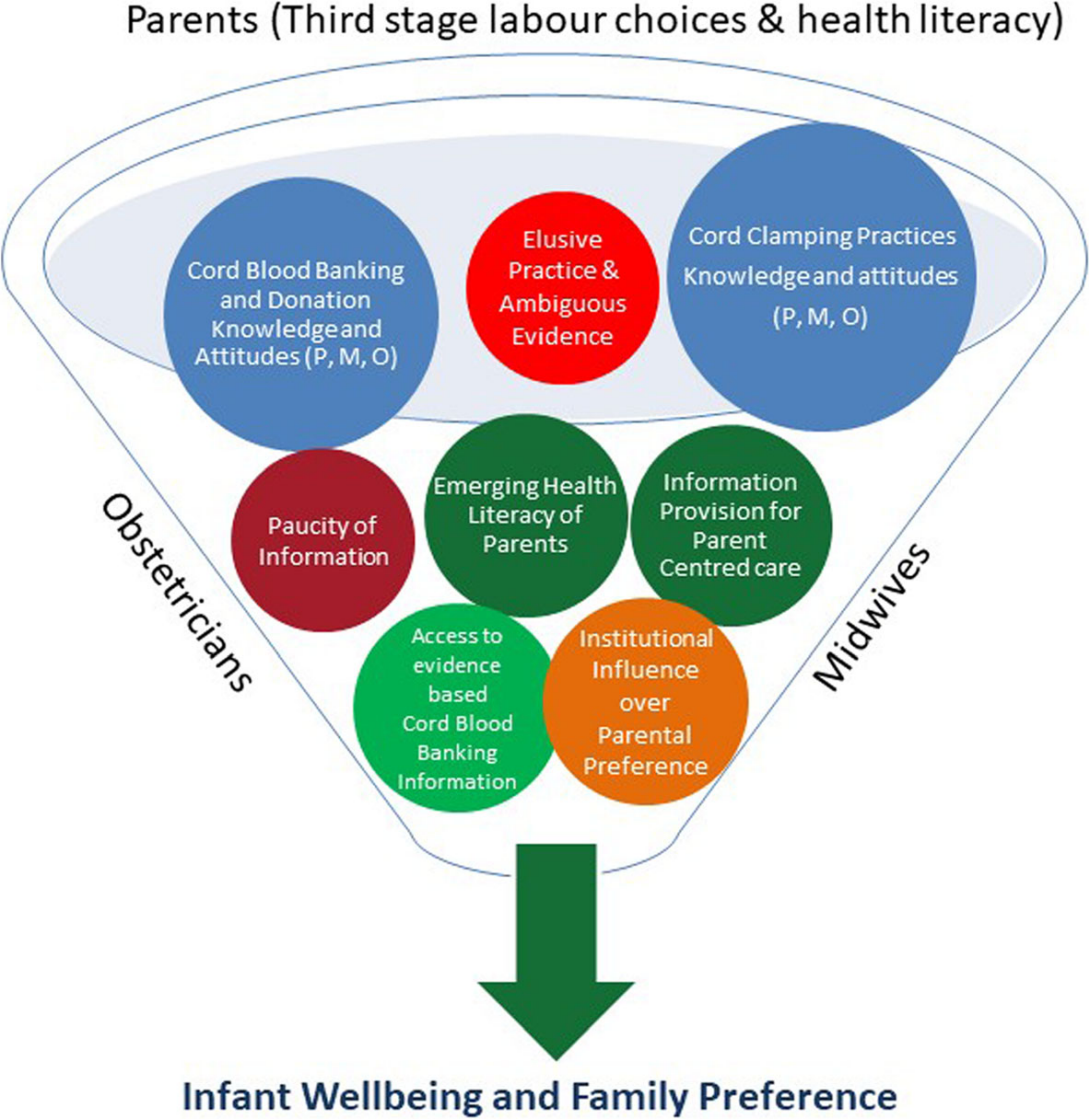
Cord Blood Options. Health Professionals Cord Blood Banking & Donation Practices. Health Professionals Cord Clamping Practices

Thematic analysis resulted in the identification of two overarching topic areas: *cord clamp timing*, and *cord blood banking (CBB) and cord blood donation (CBD)*. Several themes were developed within these topic areas and a Thematic map was developed to represent the overarching topic areas and subsequent themes. The Thematic map (Fig. 1) illustrates the importance of maternity health professionals’ practices of informing parents of their third stage labour options and increasing their health literacy. As a result of improved health literacy, parents can make informed decisions around their preferred third stage labour choice based on their infant’s wellbeing and family preference.

### Topic one: cord clamp timing

Cord clamp timing, in particular deferred cord clamping (DCC), was valued and supported by participants for parents’ third stage labour choice. Four themes were constructed from the transcribed data: Information provision for parent-centred care; Emerging health literacy; Elusive practice and ambiguous evidence; and Institutional influence over parental preference.

#### Theme one: information provision for parent-centred care

Significant value was placed on informing parents about the importance of cord clamp timing in the third stage of labour. Most midwives believed that parents should be informed about the value of placental transfusion which occurs in the minutes following birth, and this information should be embedded as part of ‘routine’ antenatal care.

*Absolutely it is important to discuss cord clamp timing, even if there is an education gap there, parents need to know the benefits of DCC versus ECC. (Midwife)*

Obstetric participants demonstrated differing levels of engagement regarding initiation and discussion of cord clamp timing with parents as part of routine antenatal and/or intrapartum care. One regional-based private practice obstetrician incorporated discussions about cord clamp timing into his antenatal discussions with parents:

*DCC is part of my routine, so it is not something that I have started doing recently. So, I tell them I routinely defer the cord clamping. (Obstetrician)*

Although most obstetricians supported DCC, one obstetric participant who worked in private practice appeared ambivalent to the practice, for example:

*I don’t offer too much negative feedback about it because they want it done and it does no harm, it is easy to do so I say fine…..I just give them the facts. (Obstetrician)*

#### Theme two: emerging health literacy

Recently there has been a clear shift in parents’ interest in and knowledge of cord clamp timing as part of labour care, particularly DCC and participants reported that parents raised this topic in antenatal consultations. Discourse reflecting parental engagement in pregnancy and birth care was noted. Parental enquiry into labour care options sometimes resulted in contrasting preferences. These were identified by participants as an interesting space for educational opportunities – especially when the preferences may not be possible to facilitate simultaneously.

*It is becoming increasingly something that people bring up……and funnily enough it is being brought up by people who are looking at doing cord blood banking. I tell them they can do one or the other but not both. Because if they want to do CBB, they need a decent amount there. (Obstetrician)*

#### Theme three: elusive practice and ambiguous evidence

Despite broad support for the practice of DCC by participants, a lack of consistency as to what constituted DCC emerged from the participants’ narrative. Cord clamp timing was dependant on the individual clinician’s perception of what constituted DCC.

*I just do DCC if the patients want it ….so if people want me to do deferred cord clamping, I will do it, but I just clamp the cord whenever. (Obstetrician)*

Some midwifery participants also identified that their practice of DCC was not guided by a specific time frame. Disparities in practice of cord clamp timing were not only evident between midwifery and obstetric participants but also identified within the disciplines. DCC practices are therefore ambiguous and dependent on individual clinicians’ perceptions or perspectives about what constitutes DCC. Evidence based practice and clear definitions were rarely used to justify practice.

Midwifery participants spoke about the requirement for more definitive and transparent information about the optimal cord clamping time. Guidelines were identified as important resources in promoting this transparency. An important gap in knowledge, understanding and evidence about cord clamp timing was evident in the transcripts.

*More solid recommendations around cord clamp timing is needed as there remains so much variation in recommendations out there. Such as some say DCC is 60 seconds, others say optimal is 1 – 3 minutes, others say wait until the cord stops pulsating so it would be good to have consistency with more clear evidence around that. (Midwife)*

Although, participants identified the need for more definitive practice guidelines related to the optimal time interval to clamp the cord, it was evident that a more holistic and patient centred approach be considered. Many midwifery participants voiced their belief that the cord should only be clamped after pulsations have ceased.

*Just that it annoys me that some health care professionals stick to the 1 – 3 minutes rigidly for DCC, when really just let the cord pulsate until the end then clamp it. I also believe it can pulsate for much longer than this time and still provide benefit to the baby. (Midwife)*

A regional based midwife working in both the public hospital system and community practice verbalised that cord clamp timing should be reframed, and that DCC needed to be regarded as normal practice.

*And I think that we need to do some more research on what the effects are of early cord clamping (ECC) because really that is the intervention, not the DCC …I think that we need to reframe how we are talking about our research. I think instead of talking about the benefits of DCC, talking about the risks of ECC and putting the physiological um back, like reframing that (deferred clamping) as being normal. (Midwife)*

#### Theme four: institutional influence over parental preference

Participants identified individual clinical scenarios as an important factor in influencing cord clamping practice, for example, when newborn resuscitation was required.

*I would never not uphold parents’ wishes for DCC, it would only be in the situation where the baby needs resus or the mother is having a bleed. (Midwife)*

DCC in the case of a compromised infant may promote better outcomes for the infant [20] and some participants were aware of this. However, DCC if the infant required resuscitation was not practised. This was often overridden in favour of traditional resuscitation processes of early cord clamping and removing the infant to the resuscitaire.

*If the baby needs immediate resuscitation then we tell parents they probably won’t have DCC although research shows this is probably beneficial to have DCC in these situations. (Midwife)*

Some midwives expressed that regardless of whether the infant appeared to be slow to respond to extrauterine life, they would still defer the clamping of the cord to uphold parent wishes for deferred cord clamping. One obstetric participant employed in the public hospital system revealed that she was also supportive of not immediately clamping the cord to allow some time to see if infants’ “pick up” while receiving the support of continued placental transfusion that deferred cord clamp facilitates. However generally, parents’ decisions about DCC when infants were slow to respond at birth were often overridden by other health professionals who reverted to ECC practices and active resuscitation.

*Um, sadly you know when there are other health professionals in the room like paediatricians or obstetricians, they override that choice of DCC and resuscitation with an intact cord. If it is just me, I would wait a minute before I clamped if it needed resus but if there are other health professionals there you don’t get to make that decision. (Midwife)*

Overall, universal support for and practice of DCC in healthy, robust infants was evident. However, in emergent clinical scenarios such as neonatal resuscitation, cord clamp timing practices varied with a feeling of uncertainty and interdisciplinary conflict apparent. Individual preferences were voiced, frequently unsubstantiated by evidence.

### Topic two: cord blood banking and donation

In contrast to the support for DCC, CBB was regarded by most participants with scepticism and suspicion. Two themes were constructed from the verbatim data: protective steering, and paucity of information.

#### Theme one: protective steering

The discourse between health professional and parent regarding cord clamp timing was identified as an important element of antenatal education by most of the participants. However, participants in this study did not see an open and transparent discussion about cord blood banking as an essential or even important part of antenatal education.

*I don’t initiate the conversation about CBB because I think it is a private thing and to be honest, it doesn’t occur to me to bring up the conversation. (Midwife)*

Obstetric participants also revealed that discussing or informing parents about CBB was not a priority or part of their routine antenatal discussions. Midwives in this study held strong perspectives about the value of cord blood, and these perspectives often underpinned the rationale for why CBB was not initiated as part of routine antenatal education and care.

*I think it is unethical to mention CBB. If there is a real reason that they need that, they would have already researched it and um I don’t want to sound like I am endorsing it in anyway. Most people don’t know all the things around DCC, but they know that it is a good thing and they do it. (Midwife)*

When parents did initiate the conversation about the option of CBB, some midwifery participants identified that this was not within their scope of practice and directed parents to do their own research.

*We would just get them google it to be honest. I don’t think I have ever seen any information on CBB or seen any brochures anywhere sort of lurking around in the hospital either. (Midwife)*

Participants used educational resources such as cord blood bank brochures to inform interested parents about CBB.

*We don’t actively promote CBB but we have brochures in our clinics. If they ask questions, we tell them to go away and do their own research. (Midwife)*

Similarly, by a regional-based obstetrician in private practice stated:

*Increasingly people are asking about it and we have literature in the rooms which we give out……… if they bring it up that is fine. I give a fairly neutral overview as I don’t want to be seen to be promoting an increased expense for parents. (Obstetrician)*

A more balanced approach to information sharing about cord blood donation was revealed. Parents make informed decisions about either DCC or CBD. A midwife who worked in a hospital where the option of CBD was available to parents stated:

*Some of them do ask about CBD. And we certainly talk about it to the women and say that it is available, it is altruistic, you are giving your cord blood for the purpose of research and it is no benefit to you but it is potentially helping other people and they use it to try and find cures for all sorts of different things. There certainly are people that are interested but I wouldn’t say it is the majority, I would say it is the majority are keen to hang onto their own cord blood. ……. (Midwife)*

#### Theme two: paucity of information

Limited knowledge of CBB and CBD was evident amongst participants.

*In regard to CBB and CBD – I don’t think any of us know anything about that. We are in the dark as much as our patients are most of the time. (Midwife)*

Yet, despite the self-identified lack of knowledge about cord blood banking or the possible benefits, participants were more likely to promote DCC over CBB.

*If someone asked me about it, I would say it is a personal choice or decision, I don’t know much about CBB so I would direct them to the internet for more information. If anyone asked me my opinion, then I would recommend DCC because there is much more evidence surrounding it than CBB. (Midwife)*

Knowledge of CBB was limited in this study and there were limited attempts to engage in professional development and increase awareness and knowledge for the benefit of parent education. Knowledge about reasons for CBB was also often inadequate or incorrect in this study, perhaps informed by media anecdotes or fictional stories.

*The only reason for CBB is if it they needed a sibling’s cord blood. (Midwife)*

Participants did not appear to be aware of the potential use of cord blood stem cells for regenerative medicine and other therapies. In contrast some participants identified a need for more information on CBB, in particular evidenced-based information.

*In regard to CBB it would be great to get some honest and unbiased information on what the benefits actually are. (Midwife)*

## Discussion

This qualitative study revealed overall strong support for DCC, and this was seen as important and routinely discussed with parents as part of antenatal care. However, this support did not extend to the options of CBB and CBD and routinely informing parents of these options when they were available at their birthing location.

The positive elements of DCC were communicated to parents by midwives. Midwives also supported DCC to be discussed as the normal cord clamp timing practice and for early cord clamping (ECC) to be reframed as the intervention. Although supportive of DCC and respectful of parental choice, obstetric participants displayed inconsistency in the level of importance placed on initiating this conversation. This finding reinforces the need for consistency of practice. To date, no studies have identified or explored the practice of informing parents about cord clamp timing as part of labour care and the new insights revealed in this study call for more standardisation of practice and transparency in relation to information sharing with parents.

Increasing health literacy of expectant parents regarding cord clamp timing during the third stage of labour was revealed. Parents receive their information from a variety of sources – media, family, friends – and often these sources can foster inaccuracies and misperceptions about options to achieve healthy birth outcomes [21]. Parents link trust with health professionals [22], therefore emphasis must be placed on assisting parents to understand and utilise all the information that is readily available to them [23].

Health professionals’ perspectives towards the practice of cord clamp timing have been investigated by several researchers [10, 12, 24]. Despite cord clamp timing guidelines recommending DCC as optimal practice [2, 3, 25], clarity and consistency about what constitutes DCC is still urgently required. This study revealed that DCC practice was often based on subjective assessment and clinician’s judgement. Without clearly defined evidence-based guidelines it remains problematic for maternity health professionals to promote and provide best practice during the third stage of labour.

Inconsistent practice was particularly evident in the case of cord clamp timing for compromised infants. Clinicians revert to ECC in the presence of a compromised infant because it is argued that this practice enables active resuscitation [12, 13, 26–30]. Participants in this study identified ECC as a priority if the infant was compromised at birth. In contrast however, evidence to date suggests that active resuscitation at the bedside with an intact cord may improve outcomes for infants requiring resuscitation [20].

Australia currently has one private and three public cord blood banks. Collection of cord blood for private banking purposes can be performed at most maternity hospitals in Australia. Collection of cord blood for donation to the public cord blood banks can be performed at eleven maternity hospitals nationally.

The practice of informing parents about CBB is not part of routine antenatal care and only provided if parents initiate the conversation [31], as maternity health professionals do not see participating in CBB activities as within their scope of practice [32]. Whereas obstetric participants in this study did not initiate conversations about CBB with parents, they would discuss and provide general information on this option to parents when prompted. However, strong personal perspectives around the value of cord blood, and opposition to CBB, were the reasons why the option of CBB was not initiated with parents and actively avoided by midwives. Midwives ‘protectively steered’ [33] parents towards the third stage option that they felt was in the best interest of the infant – DCC - through the inclusion of this information they identified as important with suppression of other information such as CBB.

Similar strong perspectives have been identified by other researchers with CBB being described by midwives as a ‘trendy, grim, useless and selfish act’ [34]. This finding is concerning because these strong and negative statements indicate that personal values and biases likely affect the presentation of evidence [22], and that midwives position themselves as advocates for the infant, and that the philosophy of woman/parent centred care and choice is not always translated into practice [35]. The practice of not providing information on all options available to parents in the third stage of labour is concerning because maternity health professionals can influence how parents’ rights to quality information during pregnancy and birth are promoted and upheld [35].

In comparison, most midwives interviewed revealed that when parents enquired about CBB, they simply referred them to do their own research. This finding is important as it is contrary to midwives’ practice of openly and transparently discussing cord clamp timing. Lack of transparency and openness about CBB creates concerning discourse about provision of evidence-based information for parental informed decision making [36, 37] because women/parents want to be informed of their options [38], and they are aware that the internet had the potential to provide ‘unreliable’ information [39].

Parents value information about CBB and their preference is that their maternity health care provider/s share this [40]. Participant narratives revealed a greater level of knowledge about the potential use of cord blood and current clinical trials is necessary if maternity health professionals are to provide parents with evidence-based information. Participants verbalised they would like this information, identifying a need for open and frank discussions with parents and being confident to respond to questions about CBB.

Request for evidence-based CBB scientific information may reflect the current information available for maternity health professionals provided by CBBs, who market CBB as an insurance policy for possible future use as opposed to evidence-based scientific focus on actual current benefits of collecting and storing cord blood [41, 42]. Informal mechanisms such as CBB literature have been identified as the most common source of information on CBB for maternity health professionals [43–46]. Informal sources may not have undergone rigorous quality assurance process, may lack governance or not been peer assessed so the validity of the content may not be entirely accurate and evidence-based.

International literature surrounding CBD demonstrates maternity health professionals’ perspectives are more positive towards this option, than CBB [34, 41, 47, 48]. CBD is regarded by maternity health professionals as altruistic and ethical [34]. The findings in this study support previous research despite participants’ low knowledge levels and minimal exposure to the option. Because CBD is an act of altruism and does not incur a financial cost to parents, this may have influenced participants’ views about this option, thus being more acceptable to discuss with parents. Some participants wanted CBD to be more accessible for parents because this was an option parents enquired about and expressed interest in during antenatal consultations.

### Strengths and limitations

This is the first study to investigate the perspectives of maternity health professionals relating to third stage labour options of cord clamp timing, cord blood banking and cord blood donation, including Australia, where access to these options is increasing. The findings from this study have provided new and unique knowledge and understanding about this complex, and inter-related aspect of care.

A further strength of this research was the use of a semi-structured interview method which allowed for flexibility throughout the interview process, yet ensured all required information was covered and collected. Flexibility allowed the researcher to explore and clarify participant responses during the interviews and for deeper exploration into participants’ perspectives and practices using prompts or further probing questions [14].

There were also limitations with this study. Most participants identified as midwives (79%), with limited representation of obstetricians. Although this research was originally intended to be national study, recruitment issues also resulted in the majority of participants being from Queensland (71%) where the researcher was based.

### Implications for practice

Study findings may contribute to the development of future education curriculum of maternity health professionals pertaining to cord clamp timing, cord blood banking and cord blood donation. Future education for maternity health professionals about third stage labour options, should aim to promote the practice of open discussion and consistent, objective, evidence-based information sharing. Provision of quality ante-natal education which includes all third stage options will allow parents and families to make fully informed decisions about their care options and be true partners in their care.

## Conclusion

This study has provided unique understanding of maternity health professionals’ knowledge, perspectives and practices pertaining to parents’ third stage labour options of cord clamp timing, cord blood banking and cord blood donation. More clarity and consistency are required in the form of evidence-based guidelines to help guide clinical practice regarding the optimal time to clamp the cord. The study has also revealed that formalised education for maternity health professionals is needed about the benefits and implications of cord blood banking and cord blood donation so that they have the confidence to openly discuss all options of cord clamp timing, cord blood banking and cord blood donation with parents. Provision of evidence-based information contributes to parent centred care and facilitates fully informed parent choices about the option that best suits their family’s circumstances, values and perspectives.

## Supplementary information

**Additional file 1.**

## Data Availability

Data were collected for the purposes of this study only and are not available beyond this scope. Data requests may be considered by the corresponding author on request.

## Abbreviations

AHPRA: Australian Health Practitioner Regulation Agency
CBB: Cord blood banking
CBD: Cord blood donation
CCT: Cord clamp timing
DCC: Deferred cord clamping
ECC: Early cord clamping
