# Supplementary material for: Maternity health professionals’ perspectives of cord clamp timing, cord blood banking and cord blood donation: a qualitative study
Source: BMC Pregnancy Childbirth. 2020 Jul 16;20:410. doi: 10.1186/s12884-020-03102-8 (PMC7364524; doi:10.1186/s12884-020-03102-8)
Supplement: Supplementary file 1 — Additional file 1. [file 12884_2020_3102_MOESM1_ESM.docx]

**Topic Guide for Interviews**

The qualitative interviews for health professionals (midwives and obstetricians) will involve questions related to their practice of informing expectant parents of their third stage labour options of cord clamp timing, cord blood banking and donation.

Specific elements are identified for each of the topics and will each broadly centre on health professionals’ attitudes and practices.

**Cord clamp timing:**

Attitudes and practice of informing parents on cord clamp timing

Examples of questions:

1. What cord clamping guidelines are in place at your workplace?
2. Do you follow them closely or use as a guide
3. Can you tell me about your experience regarding informing expectant parents about cord clamp timing? (Do they ask about it? Do they appear interested in this aspect of labour care?)
4. Do you think that it is important to discuss cord clamp timing with expectant parents? (Why? Why not?)
5. Tell me about the information you usually discuss with expectant parents about cord clamp timing? (eg: process, Delayed Cord Clamping (DCC) v Early Cord Clamping, terms that you use, eg delayed or deferred?)
6. What do you think are the important points to discuss with expectant parents about cord clamp timing? (eg: value of placental transfusion? Benefits/perceived risks of DCC & ECC?)
7. What information do you think would assist you to provide expectant parents with information about cord clamp timing to allow them to make an informed decision about this aspect of labour care? (eg: value of placental transfusion? Benefits/perceived risks of DCC v ECC?)
8. How often, in your experience, are parents cord clamping preference upheld? (eg: If there is a situation during the birth process where the parents’ clamping wishes may not be the recommended action (ie/ emergency birth), are they informed before alternate action is taken? Are they given a choice in the alternate action? Are they advised in the initial cord clamping discussion that in some situations their clamping choice may not be able to be upheld, and if so alternate action may be taken?)
9. In a normal, uncomplicated birth scenario, when do you usually clamp the cord? (eg: based on parents’ wishes? DCC v ECC?)
10. What do you think is the impact/importance of cord clamp timing on mother and on the infant and why?

**Cord blood banking:**

Attitudes and practice of informing parents on cord blood banking

Example of questions:

1. Can you tell me about your experience regarding informing expectant parents about cord blood banking? (Do they ask about it? Do they initiate the conversation? Do you initiate the conversation? What sort of questions do they ask?)
2. Do you think that it is important to discuss cord blood banking with expectant parents? (Why? Why not?)
3. Tell me about what information you usually discuss with expectant parents about cord blood banking? (if you do?)
4. What do you think are the important points to discuss with expectant parents about cord blood banking? (eg: collection process, cord clamp timing, evidence for/against CBB?)
5. What information do you think would assist you to provide expectant parents with information about cord blood banking? (eg: scientific evidence? Clinical trials? current use?)
6. Who do you think is best placed to discuss cord blood banking with expectant parents? (eg: antenatal health professionals? cord blood bank staff?)

**Cord blood donation:**

Attitudes and practice of informing parents on cord blood donation

Example of questions:

1. Can you tell me about your experience regarding informing expectant parents about cord blood donation? (Is it an option where you practice? Do they initiate the conversation? Do you initiate the conversation? What sort of questions do they ask?)
2. Do you think that it is important to discuss cord blood donation with expectant parents? (Why? Why not?)
3. Tell me about what information you usually discuss with expectant parents about cord blood donation?
4. What do you think are the important points to discuss with expectant parents about cord blood donation? (eg: collection process? What this means for cord clamp timing?)
5. What information do you think would assist you to provide expectant parents with information about cord blood donation? (eg: scientific evidence? Clinical trials? Current use? Access to the CB unit?)
6. Who do you think is best placed to discuss cord blood donation with expectant parents? (eg: cord blood bank staff, antenatal health professionals?)

Is there anything else you would like to say about cord clamp timing, cord blood banking and cord blood donation?
